# Epidural Arteriovenous Fistula After Endoscopic Lumbar Laminectomy: A Rare Postoperative Presentation

**DOI:** 10.7759/cureus.85738

**Published:** 2025-06-10

**Authors:** Hideki Hayashi, Hirokuni Hashikata, Ryohei Goda, Yoshinori Maki, Hiroki Toda

**Affiliations:** 1 Neurosurgery, Uji Tokushukai Hospital, Kyoto, JPN; 2 Neurosurgery, Medical Research Institute Kitano Hospital, Public Interest Incorporated Foundation (PIIF) Tazuke-Kofukai, Osaka, JPN; 3 Neurosurgery, Ayabe Renaiss Hospital, Ayabe, JPN; 4 Neurosurgery, Hikone Chuo Hospital, Hikone, JPN

**Keywords:** endoscopic spine surgery, endovascular treatment (evt), epidural arteriovenous fistula, lumbar spine surgery, surgical treatment

## Abstract

Spinal dural and epidural arteriovenous fistulas (AVFs) are rare. However, the presentation of epidural AVFs after endoscopic surgery has rarely been described. This article reports a rare case of an epidural AVF that presented after an endoscopic lumbar laminectomy. A 58-year-old man underwent endoscopic lumbar laminectomy at the L2-L4 level with 50 ml of intraoperative blood loss and no intraoperative complications, including bleeding, dural injury, or cerebrospinal fluid (CSF) leakage. He presented with recurrent motor and sensory disturbances in the right lower extremity. Follow-up magnetic resonance imaging (MRI) examination four months after endoscopic surgery revealed a hyperintense lesion in the lower thoracic spinal cord. Steroid pulse therapy and plasma exchange for possible myelitis failed to improve neurological deficits. During this period, motor and sensory disturbances in the left lower extremity and dysuria appeared. Repeated MRI revealed multiple flow voids ventral and dorsal to the thoracic spinal cord. Selective angiography of the right segmental L2 artery revealed an epidural AVF fed by a dorsal somatic branch. The epidural AVF drained retrogradely into the perimedullary vein. Direct obliteration of the epidural AVF was planned. The epidural AVF was successfully treated surgically. Postoperatively, the motor weakness in the lower extremities and dysuria resolved. No recurrence occurred after seven months. Epidural AVFs can present following endoscopic lumbar laminectomy. This condition should be considered a rare postoperative manifestation.

## Introduction

Endoscopic lumbar spinal surgery has emerged as a safe and effective technique for treating various spinal conditions, including lumbar disc herniation, stenosis, and foraminal stenosis. A comprehensive literature review identified dural tears, perioperative hematoma, transient dysesthesia, nerve root injury, and recurrence as the most common complications across different endoscopic approaches [1]. However, spinal epidural arteriovenous fistula (AVF) has rarely been reported as a complication of lumbar spine surgery [2-10]. The initial misdiagnosis rate of spinal dural AVF is 81%, while the erroneous treatment rate is 62% [10]. It is difficult to suspect dural AVF after lumbar spine surgery because of nonspecific manifestations. Common mimickers, such as transverse myelitis, multiple sclerosis, chronic inflammatory demyelinating polyneuropathy, postoperative scar tissue, and discitis, may present with overlapping symptoms, making early recognition of vascular lesions more challenging. It is imperative to acknowledge the potential of diagnostic delays as a contributing factor to permanent motor, sensory, and sphincter dysfunction. Therefore, it is crucial to establish a differential diagnosis to ensure comprehensive patient management.

Spinal arteriovenous shunts are typically classified based on their anatomical locations and venous drainage patterns. According to the updated classification proposed by Takai et al. [11], these lesions are categorized into five main types: dural AVF (Type I), glomus arteriovenous malformation (AVM) (Type II), juvenile AVM (Type III), perimedullary AVF (Type IV), and epidural AVF (Type V). Type V is further divided into Type Va, which drains into the intradural venous system, and Type Vb, which drains into the epidural venous plexus. This classification system provides a clinically relevant framework for diagnosis and treatment planning.

Herein, we report a rare case of Type Va epidural AVF that occurred after endoscopic lumbar laminectomy and was surgically treated. While a limited number of spinal epidural AVFs following lumbar surgery have been reported, these cases often share common clinical challenges: a highly variable latency period between surgery and symptom onset, insidious progression of motor and sensory deficits, and frequent delays in accurate diagnosis due to nonspecific clinical presentations and misleading imaging findings. Conventional magnetic resonance imaging (MRI) may fail to reveal abnormal vascular structures in the early phase, and the resulting diagnostic ambiguity can lead to delayed treatment and irreversible neurological deterioration. Moreover, the precise pathogenesis of postoperative AVFs remains incompletely understood, although the proposed mechanisms include surgical stress, venous hypertension, and angiogenesis induced by local inflammation [2-10]. In this context, the present case not only contributes to the growing recognition of this rare postoperative complication but also provides an opportunity to synthesize prior reports and highlight critical pitfalls in diagnosis and management.

## Case presentation

**Figure 1:**
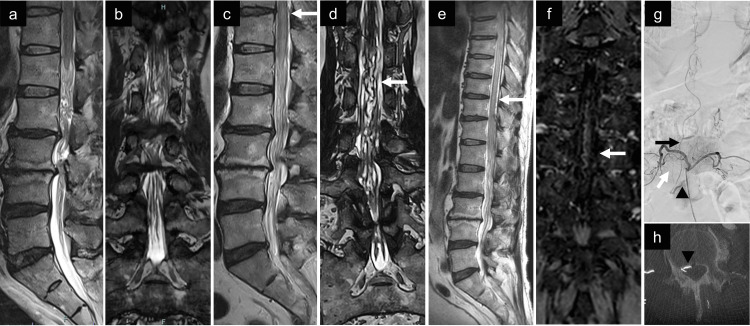
Preoperative radiological findings. (a) Initial sagittal T2-weighted MRI showing lumbar spinal canal stenosis at the L2/L3 and L3/L4 levels, with faint intramedullary hyperintensity. (b) Initial coronal FIESTA MRI showing lumbar spinal canal stenosis at the L2/L3 and L3/L4 levels, without abnormal vessels. (c) Sagittal T2-weighted MRI after laminectomy showing resolution of the spinal canal stenosis, but a slight flow void (white arrow) was observed dorsally to the conus medullaris. Hyperintense lesions in the conus medullaris were also observed compared to the initial image. (d) Coronal FIESTA MRI after showing lumbar spinal canal stenosis at the L2/L3 and L3/L4 levels without abnormal vessels (white arrow). (e) The hyperintense lesion was enlarged longitudinally to the mid-thoracic level. Multiple dorsal flow voids (white arrow) were concurrently observed. (f) Coronal contrast-enhanced MRI demonstrating an abnormal vessel (white arrow) running longitudinally in the thoracic spinal cord. (g) Digital subtraction angiography of the right L2 segmental artery, revealing an epidural arteriovenous fistula fed by the dorsal somatic branch (white arrow). The fistula (black arrowhead) drained into the perimedullary vein (black arrow). (h) The shunt pouch (black arrowhead) was located in the epidural space on the axial image of the rotational three-dimensional angiography. MRI: magnetic resonance image; FIESTA: fast imaging employing steady-state acquisition

A 58-year-old man presented with intermittent claudication and underwent endoscopic lumbar laminectomy for L2/3 and L3/4 lumbar spinal canal stenosis at another hospital in March 2024 (Figures 1a-1b). The operation was performed using a tubular retractor without pumped irrigation for 78 minutes, with 50 ml of blood loss and no complications like cerebrospinal fluid (CSF) leakage. The patient's symptoms improved during the first two months postoperatively, but gradually developed weakness and sensory deficits in the right lower extremity in May 2024. Lumbar MRI two months after laminectomy showed improvement in spinal canal stenosis, accompanied by slight hyperintensity and signal voids in the conus medullaris (Figures 1c-1d). Four months after laminectomy, the patient was referred to the neurology department of our hospital (Kitano Hospital) in July 2024 because of the rapid worsening of motor paralysis after caudal epidural block injection at another clinic. The specifics of the injection were not accessible. Lumbar puncture revealed a normal cell profile, normal protein levels, no abnormalities on flow cytometry, and negative CSF oligoclonal bands. Thoracolumbar MRI revealed hyperintensity below the mid-thoracic level on T2-weighted imaging, leading to a diagnosis of myelitis (Figure 1e).

The patient was initially treated under the diagnosis of myelitis with intravenous methylprednisolone at a dose of 1000 mg/day for five consecutive days, followed by plasma exchange therapy administered every two to three days. Subsequently, an additional course of intravenous methylprednisolone (1000 mg/day for three days) was administered. These treatments yielded only a transient and mild improvement in motor function and bladder symptoms, which gradually worsened. Contrast-enhanced gadolinium MRI revealed abnormal blood vessels on the anterior and posterior surfaces of the thoracic spinal cord (Figure 1f). Therefore, the patient was referred to our neurosurgery department in August 2024. The patient had bilateral hypoesthesia below the groin, with grade 3 strength on the right side and grade 4 strength on the left lower extremity. Knee and Achilles tendon reflexes were diminished bilaterally. He had difficulty standing and bladder dysfunction. Angiography of the right segmental L2 artery showed an epidural AVF at the right L2/3 level fed by the dorsal somatic branch, draining retrogradely into the perimedullary vein (Figure 1g). Rotational three-dimensional angiography revealed a shunt pouch in the epidural space (Figure 1h).

**Figure 2:**
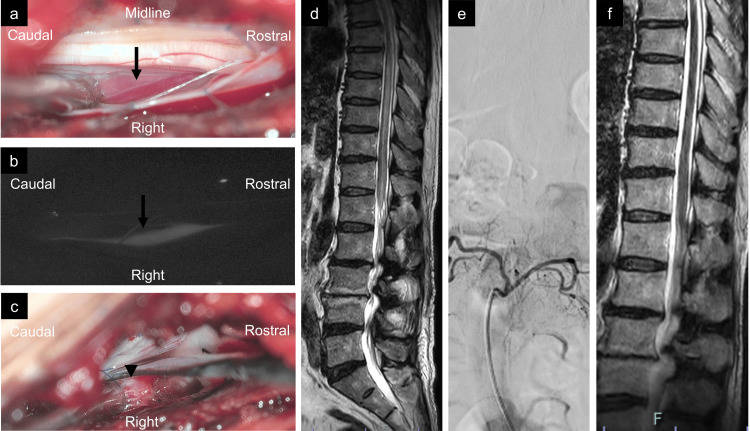
Intraoperative and postoperative radiological findings. (a,b) After opening the dura, a dilated reddish vein (black arrow) was identified. Early retrograde flow in the vein was observed with ICG (b). The veins were coagulated and obliterated. (c) The shunting pouch (black arrowhead) of the epidural arteriovenous fistula was ventrally identified after dural retraction. (d) Postoperative sagittal T2-weighted MRI showing disappearance of flow voids but with residual edema of the spinal cord. (e) Postoperative digital subtraction angiography revealing the disappearance of the epidural arteriovenous fistula. (f) Sagittal T2-weighted MRI four months after surgery showing improvement in spinal cord edema. ICG: indocyanine green; MRI: magnetic resonance image

One day after the epidural AVF was diagnosed, the patient underwent a right L2/3 hemilaminectomy and epidural AVF extraction. Owing to the urgency of the patient’s neurological decline, the surgery was performed emergently without intraoperative neurophysiological monitoring. Because a left unilateral laminectomy for bilateral decompression was performed in the previous endoscopic surgery, the adhesion between the dorsal dura and postoperative granulation needed to be dissected. However, the right lateral and ventral sides of the dura near the epidural shunt fistula were intact and the ligamentum flavum was preserved. After dural opening with an exoscope, a dilated vein emanating from the right L2/3 foramen (Figure 2a) and early retrograde filling were confirmed using indocyanine green angiography (Figure 2b). All exoscopic procedures were conducted under high magnification, and the draining vein was coagulated only after careful inspection to ensure that no critical perimedullary arteries or veins were present in the operative field. After suturing the dura, the epidural shunt pouch ventromedial to the right L3 nerve root was confirmed (Figure 2c) and was removed. Postoperative MRI showed disappearance of the dilated vessels on T2-weighted images (Figure 2d), and contrast-enhanced images and digital subtraction angiography (DSA) revealed disappearance of the epidural AVF (Figure 2e). Three weeks after the surgery, the patient was discharged for inpatient rehabilitation. His lower extremity strength improved at discharge, with grade 4 on the right side and grade 5 on the left side. From the preoperative period to three months postoperatively, Japanese Orthopaedic Association scores improved from 3 to 12 and the Oswestry Disability Index improved from 74 to 48. However, the patient continued to undergo self-catheterization for persistent neurogenic bladder dysfunction. MRI performed four months after surgery showed improvement in edema of the spinal cord (Figure 2f). At an outpatient visit seven months after surgery, motor paralysis, gait disturbance, and dysuria improved. However, numbness persisted in both lower limbs.

## Discussion

Spinal dural and epidural AVFs are rare causes of neurological deterioration after lumbar surgery. In conventional lumbar MRI, the correct diagnosis of vascular malformations is difficult because of the narrow image of the conus medullaris (Figures 1a-1b). In previous reports on AVF after lumbar surgery, almost all patients presented with severe symptoms, including bladder dysfunction (Table 1) [2-10]. Re-examination with MRI, including the midthoracic spinal cord and conus medullaris (Figure 1c), should be considered to detect spinal cord edema and dilated vessels if the patient shows clinical symptoms dissociated from the imaging findings. Given that the initial diagnosis in the present patient was myelitis due to indistinct signal voids, gadolinium contrast enhancement should be considered to reveal vascular malformations [10].

**Table 1:** Literature cases of dural and epidural AVF after lumbar surgery. ALIF: anterior lumbar interbody fusion; DSA: digital subtraction angiography; AVF: arteriovenous fistula, PLF: posterolateral lumbar fusion; PLIF: posterior lumbar interbody fusion; TAE: transarterial embolization

Author	Year	Age (years), Sex	Type of AVF	Previous lumbar surgery	Duration after lumbar surgery	Duration between treatment and symptom	Feeder on DSA	Symptoms	Treatment	Proposed Pathogenesis	Follow-up	Outcome
Yoshino et al. [2]	1998	27, Male	Dural AVM	L4-5 ALIF, L5-S discectomy	7 years	Not mentioned	Left iliolumbar artery	Motor and sensory	TAE + Surgery	Thrombus or thrombophlebitis	Not mentioned	Partial relief
Asakuno et al. [3]	2002	60, Male	Dural AVF	L5-S discectomy	2 years	1 year	Left lateral sacral artery	Motor, sensory, and urinary dysfunction	Surgery	Thick fibrous tissue	Not mentioned	Good recovery
Cho et al. [4]	2008	49, Male	Extradural AVF	L3-5 PLIF	3 years	3 months	Left iliolumbar artery	Motor, sensory, and urinary dysfunction	TAE	Vascular injury or dural tearing	6 months	Partial relief, persistent bladder dysfunction
Khaldi et al. [5]	2009	68, Male	Epidural AVF	L3-4 discectomy	2 years	2 months	Left L3	Motor, sensory, and urinary dysfunction	Surgery	Disturbance of venous drainage by epidural manipulation	6 weeks	Improving
Lim et al. [6]	2009	65, Male	Epidural AVF	L2-4 laminectomy	4 years	1 day	Right L3	Motor, sensory, and urinary dysfunction	TAE	Injury of epidural veins, predisposing to venous thrombosis	Not mentioned	Good recovery
Rangel-Castilla et al. [7]	2011	37, Male	Epidural AVF	L4-5 laminectomy	4 months	Not mentioned	Left L3-5	Motor, sensory, and urinary dysfunction	TAE	Not mentioned	1 year	Good recovery
Murakami et al. [8]	2015	69, Male	Epidural AVF	L4-5 endoscopic discectomy	8 years	10 months	Bilateral L4	Motor, sensory, and urinary dysfunction	TAE	Occlusion of epidural venous plexus and local venous hypertension	1 year	Good recovery
Elswick et al. [9]	2020	74, Female	Dural AVF	T11-Pelvis PLF	5 years	6 months	Left T12	Motor, sensory, and urinary dysfunction	Surgery	Traumatic or inflammatory mechanism	5 months	Partial relief, persistent bladder dysfunction
Ouyang et al. [10]	2021	54, Male	Dural AVF	L3-5 PLIF	8 years	7 months	Left T10	Motor, sensory, and urinary dysfunction	Surgery	Reopening of the radicular vein	14 months	Partial relief, persistent bladder dysfunction
Present case	2025	58, Male	Epidural AVF	L2-4 endoscopic discectomy	5 months	3 months	Right L2	Motor, sensory, and urinary dysfunction	Surgery		7 months	Good recovery

On spinal angiography, all segmental arteries from the upper thoracic to the iliolumbar [2,4] and sacral arteries [3] should be catheterized to accurately evaluate the entire angioarchitecture and to develop a treatment plan. For feeding arteries, the dorsal somatic branches of these segmental arteries mainly supply AVFs and single or multiple branches are important for deciding the approach route for transarterial embolization [12]. Localization of the shunt pouch in association with the vertebrae is often difficult; subsequently, rotational three-dimensional angiography is useful for identifying the dural or epidural shunt, as shown in previous cases [8,12]. Given its ability to provide precise spatial localization and improved visualization of angioarchitecture, rotational three-dimensional angiography should be strongly considered, if not routinely implemented, in the evaluation of suspected spinal vascular anomalies. This is particularly important in patients with a history of prior spinal surgery and ambiguous MRI findings, where conventional angiography alone may be insufficient to distinguish between dural and epidural AVFs or delineate complex shunt anatomy. Understanding the location of the intradural perimedullary drainer associated with the postoperative lumbar vertebrae and instruments is critical for surgical planning, and paravertebral drainage can be a candidate access route for transvenous embolization [12].

**Figure 3:**
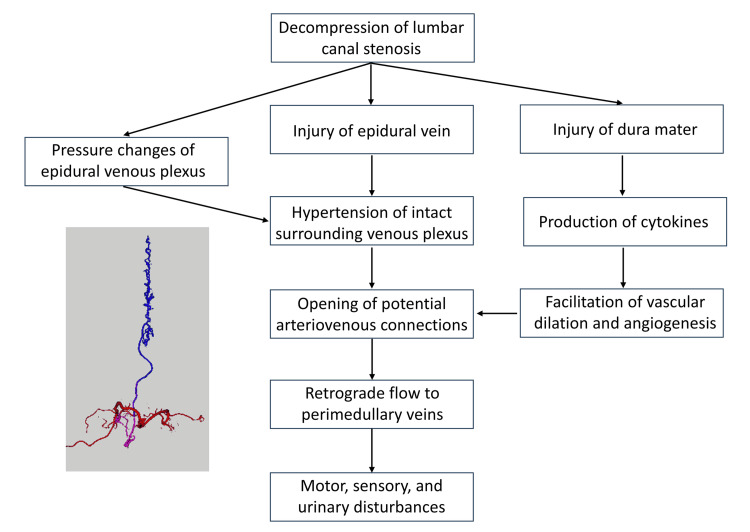
Diagram illustrating the proposed mechanism of AVF development following lumbar surgery. AVF: arteriovenous fistula The figure was created by the authors.

The mechanism of AVF is assumed to involve injury of the epidural connective tissue involving the epidural veins, predisposing the patient to venous thrombosis and leading to the formation of an AVF and complex retrograde venous drainage into the perimedullary vein [8]. In the present case, the epidural ligamentum flavum and veins were thought to be free of direct injuries on the opposite side of the surgical approach route from the previous endoscopic laminectomy. Although the duration between lumbar surgery and the onset of AVF was four months to eight years in previous reports (Table 1) [2-10], the present case showed motor and sensory disturbance only two months postoperatively. Venous hypertension caused by damage to the epidural venous plexus may have concentrated on the intact venous drainage route and induced retrograde flow to the perimedullary vein in the relatively early phase (Figure 3). In this case, the venous pressure changes caused by decompressive laminectomy may have revealed a previously existing AVF. Because local inflammation of the dura induced by surgical stress may lead to the production of cytokines, facilitation of vascular dilation and angiogenesis, and opening of potential arteriovenous connections, this mechanism may have also contributed to the development of epidural AVF in our case [13]. In the present case, histopathological analysis of the shunt pouch could not be performed because the lesion was coagulated intraoperatively to achieve complete obliteration, precluding safe tissue sampling. To date, no previous report has included histological confirmation of AVF pathogenesis in similar cases. As a result, the proposed mechanisms, such as venous hypertension, inflammatory changes, and angiogenesis, remain speculative. Further studies incorporating histopathological evaluation are needed to validate these hypotheses. Only a limited number of cases of postoperative epidural AVFs have been described in the literature; therefore, further studies should be conducted to clarify the mechanism underlying the development of postoperative lumbar epidural AVFs.

Although the patients underwent treatment after a relatively short duration of symptoms (one day to one year), 50% of the patients experienced partial relief, especially bladder dysfunction (Table 1) [2-10]. Considering the severity of permanent disabilities, prompt diagnosis and treatment are crucial. Surgical extraction of AVF is superior to endovascular treatment in terms of the degree of postoperative neurological recovery, treatment success, and recurrence rates of spinal dural AVFs [14]. According to previous lumbar surgical conditions, the fastest possible treatment is desirable because there are no significant differences between surgery [3,5,9,10] and endovascular treatment [4,6,7].

This study has several limitations. First, it is a single case report, which limits the generalizability of the findings. Second, the mechanisms underlying the development of postoperative vascular abnormalities remain hypothetical and require further investigation. Third, the follow-up period of seven months was relatively short, which is insufficient to assess the long-term outcomes and recurrence risk. In a large multicenter cohort [14], among the neurosurgical (n = 145) and endovascular (n = 50) treatment groups of single dural AVFs (n = 195), the rate of initial treatment failure or recurrence was significantly higher in the index endovascular treatment group (0.68% and 36%). Recurrence after the initial treatment, including cases identified as late as 62 months post-treatment, was documented, particularly in patients who underwent endovascular procedures. Given these findings, we recommend a minimum follow-up duration of two years, with extended surveillance of up to five years in cases treated endovascularly, to adequately monitor for recurrence or delayed complications. To address these limitations, future studies should include a larger number of patients and longer follow-up periods.

## Conclusions

Here, we report a rare case of epidural AVF that presented after endoscopic lumbar laminectomy. This case highlights the importance of considering spinal vascular malformations in the differential diagnosis of delayed postoperative neurological decline, even when the initial lumbar imaging appears unremarkable. Early recognition is critical to avoid irreversible deficits. Given the nonspecific symptoms and diagnostic delays commonly associated with AVFs, we recommend that thoracic spinal cord imaging should be considered routinely in patients with progressive neurological deterioration after lumbar surgery. Greater awareness and systematic imaging may lead to earlier diagnosis and improved outcomes.
